# “No One Does This for the Money or Lifestyle”: Abortion Providers’ Perspectives on Factors Affecting Workforce Recruitment and Retention in the Southern United States

**DOI:** 10.1007/s10995-021-03338-6

**Published:** 2022-01-08

**Authors:** Pari Chowdhary, Anna Newton-Levinson, Roger Rochat

**Affiliations:** 1 Health Equity and Rights Team, CARE USA, Atlanta, GA USA; 2 Hubert Department of Global Health, Rollins School of Public Health, Emory University, Atlanta, GA USA

**Keywords:** Abortion providers, South/Southern United States, Recruitment, Retention, Workforce

## Abstract

**Objectives:**

Movements to stem abortion accessibility and provision are underway across the southern United States. Preserving access to safe abortion requires a steady maternal health workforce. Targeted laws and limiting environments have contributed to a regional dearth of abortion providers. This study evaluates the consequences of restrictive environments for the abortion workforce to inform strategies to reduce the provider shortage in the South.

**Methods:**

We recruited twelve physicians using purposive sampling and interviewed them on their motivations and experiences practicing in the South. We employed grounded theory analysis to translate their perspectives into recommendations for provider recruitment and retention.

**Results:**

Abortion providers identified challenges relating to restrictive legislation, institutional separation of abortion from other medical services, training unavailability, safety concerns, identity struggles, and marginalization within their profession. This contributed to providers widely experiencing stigma and isolation within their work and life environments. Their motivations for practicing in the South despite these challenges included wanting to be impactful in areas of high need, combating health access disparites, and having personal ties to the region. Providers’ suggested increasing regional networking and training opportunities, creating an information clearinghouse, and offering additional compensation to better support their work. We conceptualized these findings into a framework detailing the challenges, impacts and opportunities for abortion provision in the southern United States.

**Conclusions for Practice:**

Our recommendations for provider recruitment and retention include cooperation between professional organizations, training programs, and healthcare institutions to create opportunities for training and networking and encourage abortion-supportive organizational and policy environments.

## Significance

Current literature on the impacts of abortion-restrictive environments mostly addresses service provision and access. This research approaches abortion provision through the lens of service providers exploring their perspectives on the motivations and challenges of working in the southern United States. Seeking to offer strategies to mitigate regional abortion workforce shortages, this study proposes recommendations for provider recruitment and retention in the South.

## Introduction

Nearly one in four women in the United States (US) will have an abortion by age 45 (Jones & Jerman, 2017). Abortion is an essential component of comprehensive healthcare. Access to abortion and reproductive autonomy is linked to maternal outcomes, economic success, and general well-being (Bahn et al., 2018; Ralph et al., 2019). Research measuring women’s and children’s health against state abortion restrictions showed an inverse relationship between maternal and child health (MCH) outcomes and the number of restrictions (Thompson & Seymour, 2017). States with 10 or more restrictions had the poorest MCH outcomes (Thompson & Seymour, 2017). Southern states, defined for this study as Alabama, Florida, Georgia, Louisiana, Mississippi, North Carolina, South Carolina, and Texas each met that criteria, suffering from increasingly restrictive environments.

Since 2019, five of these states have passed early abortion bans. All except Florida have banned the use of state funds for abortion or abortion-related care (Jones et al., 2019). Every state has active targeted regulation of abortion provider (TRAP) laws imposing restrictions on clinics and physicians. These include requiring abortion facilities to meet functional standards equivalent to ambulatory surgical centers and have transfer agreements, and clinicians to have admitting privileges at local hospitals (Grossman et al., 2014). Studies assessing the impact of such abortion-restrictive environments repeatedly demonstrate evidence that despite decreased availability of safe abortion, demand for abortion persists and providers often undergo increased burdens to meet it (Medoff, 2010; Mercier et al., 2016).

Protecting access to safe abortion necessitates the availability of a stable abortion workforce. Per recent estimates, for the 72 million women of reproductive age across the US, there are 1720 abortion providers (Jones et al., 2019). Intimidation and stigmatization by opponents have deterred abortion providers (Freedman, 2010; Joffe, 2010). To date, eight abortion providers have been murdered and countless incidents of harassment recorded (NARAL, 2015). Anti-abortion laws condone such environments and may drive providers away from already under-resourced areas (Freedman, 2010). Past studies cite restrictive policies, safety concerns, and professional discrimination as reasons why resident physicians (i.e. physicians who have completed medical school and are in training for a certain specialty) intending to practice changed their minds (Greenberg et al., 2012; Steinauer et al., 2008). Of the factors affecting abortion access in the southern US, a shortage of trained and willing physicians is perhaps the direst. Only 302 abortion providers practice in the region, with 93% of counties having no provider or clinic (Jones et al., 2019). Since 2010, the number of abortion providers has more than halved in Alabama, Georgia, Louisiana, Mississippi, and Texas (Jones et al., 2019; Rinehart & Woliver, 2014). In Mississippi, only one clinic remains for its approximately 584,000 women of reproductive age (Jones et al., 2019). Understanding providers’ needs and motivations to practice is central to maintaining abortion access in the South.

Existing literature on abortion-restrictive environments has focused on service provision and accessibility. The consequences of such environments for providers are less documented. This paper explores the perspectives of Southern abortion providers on the personal and professional challenges of practicing in the region to identify potential opportunities to mitigate these issues.

## Methods

This manuscript follows the COREQ criteria for reporting qualitative research. At the time of this study, the authors held the roles of researcher, project lead, and principal investigator respectively at Emory University in partnership with Planned Parenthood Southeast. This study was reviewed by Emory University’s Institutional Review Board and met criteria for exemption. This manuscript is not based upon clinical study or patient data.

Between February and May 2015, we recruited physicians who provided abortion services in the South by introducing this study at two national meetings of abortion providers and identifying those with self-expressed interest in voluntary participation. Providers were eligible for inclusion if, at the time of the study, they were credentialed and providing abortions in one or more southern states in either a hospital or clinic setting. Twelve abortion providers participated in this study. All engaged in informed verbal consent.

We developed and utilized an in-depth interview guide focused on various aspects of practice and associated implications with the goal of identifying challenges and opportunities related to abortion practice in the South. Interviews were typically an hour in length, conducted either in-person or via Skype by the primary author, audio-recorded, and transcribed verbatim. No personal identifiers were recorded.

After completing three interviews, we began an intensive open coding process in MAXQDA using grounded theory methodology to parse out and label data with codes to group similar and contrasting events. We coded discussions of potential solutions or recommendations as whole sections rather than isolated extracts and used memos to ensure that participant viewpoints were accurately represented. We then applied this initial codebook to the remaining transcripts refining it to develop new codes and draw connections. Once all interviews were complete, we created and applied additional inductive codes such as: loneliness, admitting privileges, not for money, protestors, and professional identity. After coding all the transcripts, we explored linkages between the data to identify broad conceptual categories and distilled codes into four overarching themes. Following our initial analysis, we presented results to 11 additional providers at two national conferences for validation and further reflection. We mapped our findings into a conceptual framework which we continually refined during analysis through group discussion and iterative exploration of connections between codes.

## Results

**Fig. 1:**
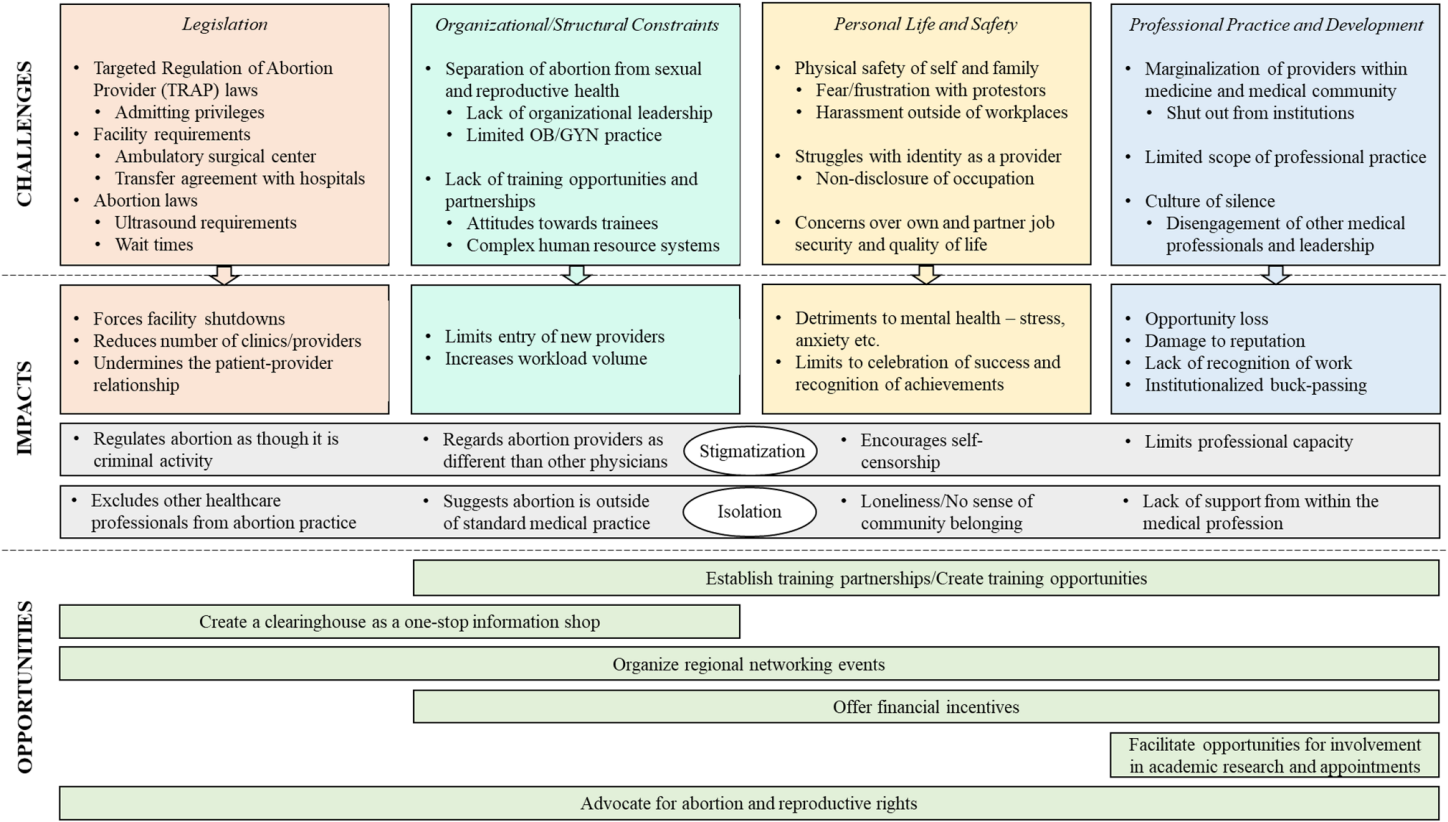
Conceptual framework summarizing the challenges affecting abortion providers practicing in the southern United States, their associated impacts, and potential opportunities to mitigate them

We interviewed seven female and five male abortion providers practicing in Alabama, Florida, Georgia, Louisiana, Mississippi, North Carolina, and Texas. Eight were under 50 years of age and seven were individuals of color. Our findings are organized into a conceptual framework illustrated in Fig. 1 and described below.

### Challenges

Abortion providers reported challenges related to legislation, institutional structures, personal life, and professional practice. As depicted in Fig. 1, each of these had unique and cross-cutting impacts on providers.

### Legislation

All providers identified TRAP laws as extremely consequential to their ability to practice in the South. Getting and maintaining admitting privileges was the most significant concern. Admitting privileges laws restricted providers’ practice by requiring them to be board-certified but providing no options or legal recourse if they are unable to obtain privileges.

“I could have a great record and still be rejected on no real grounds because hospitals don’t want to engage in abortion politics.” – Provider.

Providers identified a host of other abortion policies that affected their capacity to work including ultrasound requirements, wait times, and exclusion of other health professionals. In states that require that the same physician complete a patient’s ultrasound and the abortion procedure after the waiting period, providers described difficulties managing case volume.

“With patients all day, I can’t get to paperwork until the evening. There isn’t another provider. Staff can’t do counseling or procedures, I do it all. It’s exhausting” – Provider.

Respondents expressed that laws preventing physician assistants or advanced practice nurses from providing abortion care served to isolate abortion from medical practice.

“Puts us in a vacuum. Is any other medicine forced to operate like that?” – Provider.

Noting that assistants and nurses often receive training for and are involved in patient counseling for other areas of medicine, some providers remarked that the exclusion of advanced practice clinicians from abortion care was antipodal to the need for increased primary care access across the United States.

### Institutional Structures

Providers identified a need for healthcare organizations to mainstream abortion into sexual and reproductive health services. Commenting that they were routinely forced to choose between providing abortions and other services, providers felt their scope of practice was limited in the South.

“[Hospitals] often aren’t allies so even in spaces that are supposed to be friendly, we’re outsiders.” – Provider.

When asked what caused this perceived separation at organizations, providers identified three possible factors – undefined abortion stances of leadership or the organization, minimal provider-protective policies, and “quiet” rather than outward support from leadership. In situations where a divided work environment existed, providers said organizational leadership often determined their ability to practice.

“[Chair] has gone to bat for me. I’m not afraid of losing my appointment or having to choose, but things would be different if he wasn’t there.” – Provider.

Providers also spoke of how this separation of abortion affected their opportunities to engage in full-scale obstetrics services. They explained that often, in the interest of not engaging in abortion politics, organizations would not hire abortion-providing physicians for family care or gynecological services. One provider shared that her institution was initially supportive of her external abortion work but asked her to stop after protesters showed up, and eventually terminated her employment.

All respondents lamented the lack of partnerships between institutions and training programs in the South. Every provider identified opportunities to increase professional capacity, network, and engage in academia as fundamental to incentivizing practice.

### Personal Life

Many providers described consciously deciding whether to “come out” or keep their profession private because of concerns around backlash. Their considerations of safety extended beyond physical harm. Common elements included personal and partner job security, privacy, and quality of life. A provider shared that she had to tell her children about her work sooner than she’d hoped after protestors picketed at their school. One provider recounted receiving multiple threats.

“I was really upset when my photos were posted publicly. I can’t be confident I’m safe.” – Provider.

Providers in rural areas and Mississippi, Louisiana, and Texas reported higher frequency and severity of safety-related incidents than those in other states. While individuals’ level of concern varied by location, all expressed needing to consider either their or their family’s safety during their career. Every provider described engaging in at least one protective behavior daily. These included entering clinics through backdoors, hiding their doctors’ coat, driving different routes, and not engaging with protestors.

Despite unwavering belief in the necessity and legality of their work, all providers said they occasionally struggled with the discrepancy between how they identify with their work and how they are perceived in the environments within which they live and practice.

“I’m always having to defend what I do. Just because I provide doesn’t mean I’m never uncomfortable. There’s more to me than abortion, but that’s all I’m ever labeled as.” – Provider.

Identity struggles evolved with a provider’s length of practice, life stage, and a state’s abortion environment. Some providers said their impetus to stay out of the public eye was motivated by their family life and diminished as their children became older. Disclosure of profession ultimately affected providers’ sense of belonging, familial relationships, and emotional well-being.

### Professional Practice

Most providers believed that they experienced at least some level of marginalization within the medical community. Voicing frustrations over medical professionals being discriminatory and unsupportive, providers said this limited their practice, financial gains, and development.

“Although abortion work is necessary and challenging, I don’t command the same respect or professionalism. I’m tired of being regarded a lesser physician” – Provider.

Providers expressed that this was more deeply felt because it came from within their own profession. One provider working within a hospital setting described her colleagues as acclimatized to a culture of silence around abortion. Other providers reflected this through their concerns about the absence of medical professionals in the abortion debate.

“While we’re under attack, there is mostly silence from colleagues. When we’re applying for privileges, physicians on boards aren’t stepping up to help.” – Provider.

One long-term provider remarked that this marginalization was a consequence of the medical profession’s historical lack of endorsement and integration of abortion into standard medical care when abortion was first legalized. Providers also discussed how this translated into challenges working with healthcare professionals and finding willing support staff for abortion procedures. Specific examples included pushback from nursing and auxiliary staff on abortion paperwork.

### Impacts

Being viewed as lesser by colleagues often caused providers to feel stigmatized and working in the South further isolated them. Providers who traveled between states to provide abortions reported having to repeatedly defend their legitimacy. Others described instances of being shut out from institutions or facing discrimination by credentialing committees. Some cited examples of doctors not sharing waiting rooms with abortion patients, and staff not assisting with scheduling. Rural and sole providers for large regions reported experiencing disconnection from the medical community and concerns about having insufficient new providers to replace the workforce. Providers spoke of the valorization of their work occurring among their circles as isolating rather than empowering.

“All painting us as heroes does is make our work seem somehow exceptional and us different than other doctors.” – Provider.

They felt this disincentivized future providers by perpetuating misconceptions that abortion providers are alone, doing impossible work, and that abortion is outside of standard medicine. Providers claimed Southern state governments’ “endorsement” of such anti-abortion sentiments through abortion-restrictive legislation further impacted them by exposing them to harassment and creating a climate of fear.

Outside work, participants struggled with whether maintaining professional privacy perpetuated abortion stigma by reinforcing community perceptions that abortion providers are immoral and illegitimate. In choosing self-censorship, providers experienced feelings of loneliness and stress. One provider lamented not being able to celebrate professional successes with her social networks. Respondents also highlighted the cumulative effects of living in abortion-averse environments suggesting that stigmatization and isolation at multiple levels contributed to adverse mental and emotional health consequences.

### Opportunities

Providers’ discussions of opportunities were often related to their motivations to practice in the southern United States. Providers saw abortion as part and parcel of women’s healthcare and shared a desire to bring about change to the state of reproductive health in the South.

“It’s not a career; It’s a commitment to a cause.” – Provider.

The South’s high need for abortion services strongly affected providers’ motivations, with every participant naming the regional dearth of abortion providers, access and income disparities within already underserved populations, and an interest in making a difference as influential to their decision to practice in the South. Summarized in Table 1, providers shared potential strategies to mitigate existing challenges and the dearth of abortion providers in the South, including training partnerships, an information clearinghouse, regional networking, research, and abortion advocacy. Each of these are depicted in our conceptual framework such that they fall under the challenges that they may offset.

**Table 1:** Summary of abortion providers’ suggestions on opportunities for recruitment and retention of abortion providers in the southern United States

Description of opportunity	Provider-identified benefits
*Establish training partnerships* or *create more training opportunities* for new providers, including: Relationships between OB/GYN departments and training programs Fellowship and residency opportunities	“*Bring new providers to the South*” “*Combat declining number of providers*” “*Improve standardization of care practices if everyone is getting same training*” “*Resource sharing and greater investment in abortion provision*”
*Creation of a clearinghouse* that could: Facilitate/assist with licensing and credentialing application processes Maintain up-to-date information on a reserve pool of providers for times of scheduling conflicts Develop standardized compensation models for abortion providers	Serve as a *“regionally-specific concierge-type service”* for providers *“Free us up from some of the paperwork for licensing”* *“Connect us to be able to put out a call to other providers if we need a shift filled”* *“Help make licensing, compensation and other things cost-neutral”*
*Regional networking events* that could include: Tri/biannual regional provider meetings held within the South Psychosocial workshops	*“Just talking to other providers would be so helpful to refill our cups”* *“Mentorship for newer or isolated providers”*
*Financial incentives* that could comprise: Malpractice coverage and/or guarantees Loan repayment for newer providers Electronic privacy management services	*“Malpractice quotes are either astronomical or we’re outright denied. We need coverage”* *“Incentivize new providers to the South”* *“Cover costs of internet sweeps for privacy and reputation protection”*
Opportunities for provider participation or *involvement in academic research* post completion of residency training	*“I care about research and want opportunities to continue to carry out or engage in studies”* *“Incentivize providers to work here”*
*Abortion advocacy and deal-making* by professional organizations to: Engage pro-choice legislators Inform public about provider challenges	*“Bring in people who can lend heavy weight to the fight for abortion rights”* *“Approach the issue from all angles”* *“[Hopefully] bring about legislative change”*

Motivated by a passion to actualize the right to healthcare for women, providers repeatedly cited training as crucial to their professional capacity and satisfaction by facilitating engagement in academia, professional development, and linkages to networks. Noting that an influx of new providers could alleviate case volume and expand safe provision, providers said offering local training opportunities was critical for ensuring a continued abortion workforce in the region. To alleviate workforce shortages, providers suggested organizations create systems that allocate training time and space and improve attitudes towards trainees. Providers also suggested creating a clearinghouse to serve as a one-stop-shop for information and assistance with navigating legislative changes, licensing and credentialing, case paperwork, malpractice coverage, and scheduling.

Providers frequently discussed the importance of networking for improved support and connections to opportunities. They noted the role of professional organizations like Physicians for Reproductive Health, National Abortion Federation, and Planned Parenthood in facilitating provider networks. While providers felt that maintaining multiple memberships was cost-prohibitive, they described professional organizations as sources of support, and believed that more collaborative partnerships between them would greatly influence abortion provision in the southern United States.

## Discussion

For abortion providers in the South, restrictive legislation, lack of training, potential for harassment, and institutional marginalization levy undue burden with profound effects on their livelihood and well-being. These challenges limit providers’ scope of practice, professional development, and financial gains creating a constellation of unfavorable conditions that affect providers’ motivations to work in the South. Concerted collaboration between institutions to offer training and networking, mainstream abortion into medicine, and advocate for abortion access is crucial to help providers effectively navigate the South’s abortion restrictive contexts.

Our findings on the personal and professional consequences for providers working in the South complement existing literature. Providers’ reports of the influence of TRAP legislation on their ability and desire to practice support previous study findings that abortion laws place undue burdens on providers (Greenberg et al., 2012; Mercier et al., 2016). In surfacing the importance of training, networking, and research opportunities for their professional satisfaction, providers echoed other researchers’ conclusions that overcoming abortion provision barriers requires supplementing clinical training with networking and mentorship (Summit et al., 2020). Providers’ decision-making to disclose their profession and stories of the associated fear and burden reflect Harris et al.’s conceptual model on the dynamics of stigma in abortion work (Harris et al., 2011). Our findings that disclosure or silence are both accompanied by varying levels of coping and consequences support their hypothesis that providers undergo sustained burden of identity negotiation in highly stigmatized environments. By forcing abortion provision into only free-standing clinics, southern states have isolated abortion from standard medical practice. Providers’ experiences of marginalization and othering reinforce Harris et al.’s legitimacy paradox wherein abortion providers are regarded differently despite equivalent medical training and qualifications (Harris et al., 2013).

Providers’ experiences of stigmatization by individuals, communities, medical institutions, and state laws provide evidence of pervasive stigma towards abortion professionals in the South. Previous literature posits that abortion stigma compounds at each of these levels (Kumar et al., 2009). Our findings corroborate this—individual leaders’ support of abortion providers is limited by fear of community backlash, which is facilitated by institutional stances, which are determined by state abortion policies. Overcoming abortion stigma within medicine requires further exploration of this tension between individual views and institutional structures (Joffe, 2014). Institutions can greatly influence the stigma generated by state policies like TRAP laws that undermine the credibility and expertise of abortion providers. As demonstrated in our results, admitting privileges are difficult for abortion providers to obtain in states that require them because hospitals shy away from affiliating with abortion clinics. Often the minimum number of annual admissions that hospitals require providers seeking admitting privileges to have is higher than most providers meet since abortions rarely result in hospital visits (Fuentes & Jerman, 2019). Another example of sustained abortion stigma within medical institutions is the lack of training opportunities for providers (Joffe, 2014). By adopting supportive policies and prioritizing training, institutions can contribute to reducing the stigma that abortion providers experience in the South.

Our results have larger implications for maternal and child health in the South. Southern states have the highest rates of maternal and infant mortality in the US. Alienation of abortion providers within obstetrics and their exclusion from other service provision results in fewer available providers to deliver quality maternal health care in these areas. Our analysis showed evidence for slight variations in abortion contexts within the South. Atlanta and South Florida were less restrictive than the rest of Georgia and Florida. This study contributes a unique geographic and population focus on Southern provider perspectives to the literature, directing attention to the abortion workforce as a means of ensuring continued access to abortion and maternal health. Informed by our analysis of providers’ feedback, we identified a set of overarching recommendations that could contribute to improved provider support, recruitment, and retention in the South:

- Networking opportunities: Establishing a calendar of regional meetings with a network of abortion providers for conversation exchange and guidance could facilitate connections to reduce provider disconnect and isolation. Providers with more southern experience could mentor newer providers and possibly assist with the development of individual coping strategies.
- Training: Offering opportunities for provider training could alleviate challenges of case volume and professional isolation. Providers’ location of practice is related to their geographic location of training (Greenberg et al., 2012). Establishing structures and partnerships for more training opportunities across the South could ensure a continued workforce.
- Cooperation between organizations: Collaboration between professional associations, service providers, and advocacy groups on issues affecting abortion providers is necessary to appropriately meet providers’ needs. Such coordination could facilitate resource-sharing for regional service provision and workforce empowerment and lend a united pro-choice voice to the abortion debate.
- Abortion advocacy: By engaging in local and national advocacy work, such as developing partnerships to facilitate community discussions on reproductive rights or convening legislators to encourage pro-choice attitudes, professional associations and health agencies could contribute towards the creation of abortion patient- and provider-supportive environments in the South.

This study is limited by its small sample size. We worked to offset this by validating our findings with additional providers at national conferences.

With states and institutions adopting increasingly abortion-restrictive policies and practices, the future of abortion provision in the southern United States remains precarious. A limited workforce has significant implications for access to safe abortion and larger maternal and reproductive health outcomes. Despite the constraints that southern environments present, this study highlights the strong commitment of providers who practice there. By complementing providers’ professional motivations with supportive systems and advocacy, we can address structural barriers to affect change for the Southern abortion workforce perhaps incentivizing provider recruitment and retention in the region. For without abortion providers, there can be no safe abortions.

## Declarations

### Availability of Data and Materials

The data collected and analyzed for this study are available on reasonable request from the corresponding author (PC).
